# Addressing ethical challenges of disclosure in dementia prediction: limitations of current guidelines and suggestions to proceed

**DOI:** 10.1186/s12910-020-00476-4

**Published:** 2020-05-11

**Authors:** Zümrüt Alpinar-Sencan, Silke Schicktanz

**Affiliations:** Department of Medical Ethics and History of Medicine, University Medical Center Göttingen, Humboldtallee 36, 37073 Göttingen, Germany

**Keywords:** Biomarkers, Clinical guidelines, Communication, Dementia prediction, Disclosure, Risk information, Stakeholders

## Abstract

**Background:**

Biomarker research is gaining increasing attention focusing on the preclinical stages of the disease. Such interest requires special attention for communication and disclosure in clinical contexts. Many countries give dementia a high health policy priority by developing national strategies and by improving guidelines addressing disclosure of a diagnosis; however, risk communication is often neglected.

**Main text:**

This paper aims to identify the challenges of disclosure in the context of dementia prediction and to find out whether existing clinical guidelines sufficiently address the issues of disclosing a dementia diagnosis and of disclosing the risk of developing dementia in asymptomatic and MCI stage. We will examine clinical guidelines and recommendations of three countries (USA, Canada and Germany) regarding predictive testing and diagnostic disclosure in dementia and Mild Cognitive Impairment (MCI) to show their potentials and limits. This will provide a background to address ethical implications of predictive information and to identify ways how to proceed further. We will start by examining the guidelines and recommendations by focusing on what there is already and what is missing regarding the challenges of disclosing dementia prediction and MCI. Then, we will highlight the novel ethical issues generated by the shift to identify preclinical stages of the disease by biomarkers. We will argue for the need to develop guidelines for disclosing a risk status, which requires different considerations then disclosing a diagnosis of dementia. Finally, we will make some suggestions on how to address the gap and challenges raised by referring to German Stakeholder Conference, which presents us a good starting point to the applicability of involving stakeholders.

**Conclusions:**

This paper underlines the need to develop empirically based guidelines that address the ethical and social strategies for risk communication of dementia prediction by genetic as well as non-genetic biomarkers. According to our analysis, the guidelines do not address the new developments sufficiently. International efforts should aim for specific guidelines on counseling, communicating risk and disclosing results. We argue that guidelines on (risk) disclosure should be developed by involving various stakeholders and should be informed by socio-empirical studies involving laypersons’ needs and wishes regarding risk communication.

## Background

Dementia has recently gained much attention. Current research focuses on detection of the early phases of the disease, with a shift from cure to prevention and prediction. This shift has led to “new dementia”; that is, a reconceptualization of dementia/Alzheimer’s disease (AD) as a *continuum* with a long asymptomatic, preclinical phase characterized by slowly progressing pathological changes [1–3]. The inclusion of biomarker measures, which detect pathological changes in the brain, has led to a redefining of the earliest stages, namely the preclinical states, of AD and the disease’s diagnostic criteria [1]. This preclinical phase might convert into a symptomatic stage involving Mild Cognitive Impairment (MCI) and develop into a clinical syndromic disease [1].

Parallel to such developments in research, the growing emphasis is on effective dementia prevention through lifestyle interventions. The latest World Health Organization’s (WHO) Guidelines emphasize the importance of modifiable risk factors by targeting adults with normal cognition and MCI [4]. They stress the need to develop guidelines and recommendations to improve care, support, prevention and treatment. This shift generates novel ethical issues concerning disclosing the risk of developing dementia in the asymptomatic and MCI stages [5, 6]. Therefore, a leading research question for us was to clarify whether and how this new development of predicting dementia (e.g. by disclosing and communicating risk status) is and should be integrated in existing attempts to develop worldwide-adopted dementia strategies. For this purpose, we examined exemplarily three Western countries’ strategies and practical guidelines (the US, Canada and Germany) to show potentials and limits in existing guidelines. They demonstrate that there is a gap in existing policy frameworks, which should be addressed in close future. Therefore, we suggest a general approach concerning how ethical and social strategies for risk communication on dementia should be empirically developed and integrated in the ongoing process of dementia guidelines and national strategies.

## Main text

### An overview of current policies from USA, Canada and Germany regarding disclosure practice: what is there?

In May 2017, the 17th World Health Assembly endorsed the global dementia action plan [7]. It includes seven main targets for engagement at policy-making level, raising awareness for dementia-friendly societies, prevention for risk reduction and diagnosis, research, care and treatment of dementia. It urges its member states to develop and adopt national dementia strategies to cope with the challenges of the disease and to provide necessary care and support, both for people with dementia and for their caregivers and families, by 2025.^1^

In the following, we examine official clinical guidelines from the US, Canada and Germany. We selected these three Western countries exemplarily as a systematic review of all existing worldwide guidelines is beyond the scope of this article. However, these three countries set a leading example among Western countries where an intensive debate and research activities around dementia biomarker and prevention is well documented.^2^ The US already developed a national dementia plan (NDP) in 2011, while Canada just recently adopted one in 2019, and Germany is still in the development process. Furthermore, these three countries represent different health care systems. The US has a largely privatized system, while Germany is financed socially. Canada has a publicly financed health care system, but is also strongly influenced by the US health and research policy. In the following, we briefly summarize what is recommended regarding predictive testing, disclosure of the diagnosis of dementia, as well as of MCI and the use of biomarkers.

### The US – the new focus is on biomarkers and preclinical stages

Since 2011, the USA has had an NDP, with objectives including the creation of a dementia-friendly society, improvement of early diagnosis, care and support, as well as acceleration of treatments and prevention options [8]. The Alzheimer’s Association (AA) and the National Institute on Aging (NIA), an agency of the U.S. National Institutes of Health (NIH), jointly issued updated guidelines focusing on early detection and diagnostic accuracy [9].

#### On disclosing a dementia diagnosis

No *specific* guidelines exist regarding *how* to disclose a diagnosis. The Gerontological Society of America [10] has published a toolkit for primary care providers, which refers to Alzheimer’s Association and non-profit collaborations and organizations’ (i.e. ACT on Alzheimer’s, Group Health Cooperative) suggestions for disclosing a diagnosis. In the updated National Plan, failure to disclose a diagnosis to the persons or their families and not providing enough counseling or support is declared as a problem [8]. Hence, among the strategies, the need to educate the physicians and care providers is stressed [8].

#### Recommendations on predictive testing to determine the risk of developing AD

Biomarkers are not yet recommended for clinical diagnosis; however, practitioners can direct the persons with MCI to research centers [11]. Persons with MCI should be serially monitored for changes in their cognitive behavior, which will in turn change the available treatment options and counseling strategies [11]. Clinicians should inform the person with MCI about the limits of preventive options, but should recommend weekly exercise to improve cognition [11]. Clinicians should also inform and educate them and their families about the uncertainty of diagnosis and long-term planning, including topics such as advance directives [11].

The NIA-AA working group published recommendations on predictive *genetic testing* for APOE (Apolipoprotein E) variations, in which the importance of pre- and post-genetic counseling for both tested persons and their families is highlighted [12]. For asymptomatic individuals, the group did not recommend APOE predictive testing due to its predictive uncertainty, but it can be used as an adjunct to other diagnostic tests for AD for persons with AD. In disclosing genotype predictive test results, it is recommended that privacy and confidentiality must be respected in order to avoid adverse psychological effects, as well as unfavorable effects on insurability and employability. Later, the Standford Program in Genomics, Ethics, and Society’s (PGES) Alzheimer Disease Working Group added that predictive and diagnostic genetic testing can only be appropriate for persons with a family history of AD, and particularly for those having early-onset symptoms [13]. In addition, they recommended discussing the options and implications for genetic testing of highly penetrant genetic variations (such as PSEN1 – Presenilin-1, PSEN2 – Presenilin-2, APP – Amyloid Precursor Protein) in high-risk families while providing pre- and post-counseling.

Guidelines that are more recent emphasize the tested person’s right of choice to be tested, although genetic susceptibility testing is not clinically recommended or limited to persons with early-onset autosomal dominant AD [14]. Disclosing the results to symptomatic individuals should be both in person and in the presence of a family member accompanying the person for the tested person’s genetic counseling visits [14].

### Canada – importance of involving stakeholders becomes apparent

Very recently, in June 2019, Canada developed its national strategy. The Minister of Health worked on developing a national strategy by establishing a ministerial advisory board on dementia in 2018 and by organizing a national dementia conference, which brought together many stakeholders. The advisory board and conference participants addressed the topics of improving care and support, supporting research and innovation, educating the public by raising awareness, and reducing stigma [15, 16].

#### On disclosing a dementia diagnosis

The recommendations based on the Canadian Consensus Conference on the Diagnosis and Treatment of Dementia (CCCDTD) point out the benefits and likely harm and recommend informing the persons diagnosed with dementia and their families of their diagnosis by including information on diagnostic uncertainty, support, treatment options and future life planning [17].

In the National Dementia Conference, a number of challenges, such as the heterogeneous level of training among health care providers, lack of support and compassion when disclosing the diagnosis and clinicians’ reluctance to inform the people with dementia and their caregivers were identified [15]. Difficulty in understanding the information and lack of evidence-based guidelines^3^ as well the need for culturally sensitive guidelines and information was raised, too (i.e. more ethno-cultural data is needed to understand the impact of dementia, stigma leading to under-diagnosis and under-reporting) (see: [19]).

#### Recommendations on predictive testing to determine the risk of developing AD

For clinical diagnosis of AD or MCI, reference to biomarkers (amyloid test results) is not recommended [18]. Due to their risk of developing dementia, persons with MCI should be followed up carefully, but those persons “should not be labeled as having early AD” [20]. The family physician should encourage such persons to implement life planning and a healthy lifestyle [20].

For asymptomatic individuals, APOE screening for cognitive impairment is not recommended [17]. If a cognitive decline is reported by the family, cognitive assessment and regular follow-up should be indicated [17]. The family physician should direct the asymptomatic person with a family history suggestive of autosomal dominant inheritance to a genetic clinic; otherwise, the individual could be directed to advocacy organizations for assistance [17].

### Germany – the need to develop empirically driven guidelines on (risk) disclosure emerges

In 2012, the German government, in cooperation with the German Alzheimer’s Association, founded the national “Alliance for People with Dementia”^4^ [21]. In 2014, the alliance issued an agenda defining the “fields of action”, which was the first step towards implementing a national dementia strategy [21]. The revised version of the German clinical guidelines for dementia, *S3 Guidelines “Dementia”* [22], was jointly presented on January 27, 2016 by the two leading medical associations, the German Society for Neurology (DGN) and the German Society for Psychiatry and Psychotherapy, Psychosomatics and Neurology (DGPPN). These are the only existing, comprehensive clinical guidelines on dementia in the German context that provides clear recommendations for physicians and therapists [22].

#### On disclosing a dementia diagnosis

According to the *S3 Guidelines “Dementia”*, there must be a continuous counseling process, and the changing needs of people with dementia and family caregivers should be considered. If an individual is diagnosed with dementia, then in addition to diagnosis and information on possible treatment options, information on assistance and support services, the benefits of health and long-term care insurance and social assistance should be provided to those affected and associated relatives. Consulting on these mentioned areas is stated to be a multi-professional task [22].

#### Recommendations on predictive testing to determine the risk of developing AD

The German Medical Association (GMA) [23] published currently a statement on predictive testing to determine the risk of developing dementia. It focuses on three population groups: asymptomatic persons without a family history of AD, asymptomatic persons with a family history of AD, and individuals with subjective cognitive decline. Predictive testing for asymptomatic individuals without a family history of AD due to limited validity of the tests and lack of preventive measures is not recommended. The practitioners should carefully counsel the individuals about the possible tests and their limits. It is also not recommended to do predictive genetic testing for persons with subjective complaints. For those affected individuals, the practitioners should carefully assess their medical history, and a differential diagnostic examination is needed to exclude other plausible causes. For the asymptomatic persons with a family history of AD, there is an increased risk of developing a hereditary (autosomal dominant) form of AD. In terms of future planning, reducing fears and joining clinical trials, it is feasible to have a genetic test carried out to determine the genetic variations (PSEN1, PSEN2, APP) for those with an early-onset AD only with appropriate genetic counseling [22]. Predictive genetic testing for APOE variations (ApoE4 – Apolipoprotein E4) is not recommended for late-onset AD, as the associated predictive value in diagnostics is very limited [22, 23].

#### Future considerations for disclosing MCI diagnosis and preclinical stages

The current clinical guideline [22] does not provide detailed recommendations on risk assessments, diagnosis of MCI or the applications of biomarkers. It is stated that a procedure that could be valid for biomarker-based prediction of AD at the stage of MCI has yet to be established. Biomarker-based testing in clinical practice is acknowledged to increase the likelihood of indicating that an MCI is due to AD [22], but should be left to dementia experts. It is recommended that if a person with MCI asks for risk assessment, the physician can direct the persons and their families to research centers and they should be informed about treatment options, scientific limitations of the tests and the likely psychosocial burdens [22].

Additionally, a nation-wide German stakeholder conference focusing on conflicts in predictive dementia diagnostics took place in Göttingen in June 2018. A multidisciplinary^5^ joint statement was formulated and embedded in a larger discourse event including online commentaries^6^ [24]. The focus of the joint statement is on asymptomatic individuals, as well as on individuals with MCI. It made a strong point on how to improve guidelines for disclosing the biomarker test results for prediction for asymptomatic patients and for persons with MCI, once the certainty in prediction has substantially improved (see below). It is, however, unclear whether the current development of the national dementia plan will take up the stakeholder recommendation because, to date, its agenda touches the topics of disclosure and prediction only slightly.

### What to learn and how to proceed further? An analysis

In the following part, we discuss three main foci respectively:

- What can we learn from the existing policy strategies (such as guidelines, NDP or stakeholder discourse) regarding the challenges of disclosing dementia prediction and MCI?
- In how far are challenges of dementia diagnosis and dementia risk disclosure similar or different, and what are the implications of this comparison?
- Which directions do seem promising to address unsolved challenges of predicting dementia?

#### Questioning guidelines: what can we learn?

Our exemplary analysis illustrates that there is a gap in guidelines and policy regarding disclosing dementia prediction. The referred countries’ guidelines are raising quite similar points, but also show some differences. Very generally, in the US, there seems to be more emphasis on research with biomarkers and preclinical stages. Canada and Germany stated the importance of stakeholders’ involvement (i.e. people with dementia, their caregivers and families, academic and civil society representatives, health care professionals, regional health authorities and local networks) in developing the strategies and guidelines (see also: [7]). As the shift from research area to clinical practice increases, both countries stress the importance of developing evidence-based guidelines on disclosure practice. The challenges and concerns regarding the disclosing of a diagnosis and even risk prediction have been raised on a general level by all the three referred countries. However, except for the recommendations from the US and the general emphasis on counseling by all three countries, we could not identify any *specific clinical guidelines* regarding *how* exactly to disclose a diagnosis and risk status.^7^ Only in Germany we found a specific initiative concerning disclosing a risk status, which we will discuss in more detail below.

**Table 1:** An overview of guidelines and recommendations on disclosure processes and genetic and non-genetic predictive testing

Guidelines/ Recommendations on	Countries					
	The US		Canada		Germany	
	**Type**	**Content**	**Type**	**Content**	**Type**	**Content**
**Disclosure of Dementia Diagnosis**	Recommendations	How to disclose a diagnosis of dementia Disclosing to the tested person and to the family member Information on support, disease, treatment, care Counseling as a multi-professional task	Recommendations [Guidelines]	Disclosing to the tested person and to the family member Information on support, disease, treatment, care Diagnosis and management is the responsibility of primary health care	Guidelines	Information on support, disease, treatment, care Attention to the changing needs of the tested person and family caregivers Counseling as a multi-professional task
**Disclosure of MCI Diagnosis**	Guidelines	No specific information Importance of counseling (tested individuals and family) on lack of proven dietary or pharmacologic means, uncertainty of diagnosis, long-term planning	Recommendations [Guidelines]	No specific information Importance of counseling (tested individuals) on life planning and healthy lifestyle	Guidelines & Recommendations	No specific information Counseling with family members Input by Stakeholders Conf. to improve guidelines [Counseling should be multidisciplinary]
**Use of Biomarkers/Predictive Tests – Risk Assessment**	Guidelines	Not recommended for clinical diagnosis Individuals with MCI should be monitored If person with MCI asks for risk assessment, physician can direct families and the person to research centers Physician should inform the individuals on scientific limits of tests	Recommendations [Guidelines]	Not recommended for clinical diagnosis Individuals with MCI should be monitored	Guidelines	Not recommended for clinical diagnosis If person with MCI asks for risk assessment, physician can direct families and the person to research centers Physician should inform the individuals on scientific limits of tests, possibilities and treatment options Handling of biomarkers is left to physicians
**Predictive Testing (Genetic)**	Guidelines & Recommendations	Not recommended for asymptomatic individuals Disclosing APOE: respect for privacy and confidentiality Recommended for persons with family history & early onset symptoms Disclosing to symptomatic individuals with a family member	Recommendations [Guidelines]	Not recommended for asymptomatic individuals, but they can be directed to advocacy organizations For asymptomatic individuals with family history cognitive assessment & regular follow-up should be indicated (Physician should direct the individuals to a genetic clinic)	Guidelines & Recommendations	Not recommended for asymptomatic individuals & with subjective cognitive decline Recommended for asymptomatic individuals with family history & early onset symptoms (Counseling required) APOE is not recommended for late onset AD

Current voices recommend not testing asymptomatic individuals, but it is partly done in the research context. Furthermore, there appear more and more media reports about new biomarker tests for predicting dementia even 20 years before onset of the first symptoms [25, 26]. It is likely that concerned individuals (with or without family history) ask therefore more and more practitioners for testing, even if paid out of pocket.^8^

Furthermore, the importance of counseling before testing^9^ becomes crucial to ensure that the affected persons understand the potential consequences and potential scientific limitations of such testing [29], since the practice of ‘early’ or even predictive diagnosis is increasing and entering everyday clinical practice. A recent study held in Germany showed that there is a great heterogeneity among practitioners in interpretation, application and disclosure of biomarkers to tested persons [29]. This is because standards for disclosing a very early diagnosis of AD or a diagnosis of MCI or even risk information is lacking.

As examined, the referred guidelines on dementia and MCI emphasize the importance of counseling and family involvement in disclosing a dementia diagnosis and an MCI diagnosis. The next crucial question is, whether and how these two conditions, namely counseling and family involvement that are common for dementia diagnosis, could be transferred to be used specifically for disclosing a risk status.

#### Ethically reflecting on the difference between disclosing a dementia diagnosis and dementia prediction

There is to our understanding an important difference between a definitive diagnosis and a risk assessment. Whereas the former stands for actually having the disease, the latter presents a probability, a status of being likely to develop the disease, but not necessarily. For instance, whereas a diagnostic test informs and confirms the person having signs and symptoms of a disease, predictive genetic test informs the asymptomatic person about the likelihood of developing the disease in the future [30]. This difference of having and being at risk generates the need for different strategies for telling and receiving a diagnosis, and for risk assessment at two different levels.

Disclosing and receiving a diagnosis of AD raises ethical challenges and concerns due to the related possible outcomes, such as the attached burden of stigma and the disease’s irreversible progress [31]. Arguments supporting diagnostic disclosure of dementia are founded on promoting trust and respect for autonomy, which allows life planning in terms of finance and care, settling family matters, getting support and psychological preparation [32, 33]. However, due to the lack of an effective cure, receiving the diagnosis could be distressing for the person with dementia and their families as well as disclosing a diagnosis for a clinician [34, 35]. A study conducted with family physicians from various provinces of Canada reported that there are barriers, such as diagnostic uncertainty and complexity of the disease, which hinders the family physicians from disclosing a diagnosis, timely diagnosis and management of care [19]. Although autonomy of the person and possible benefits of disclosure are acknowledged, in practice most clinicians tend to avoid disclosing the diagnosis [31, 36]. Additionally, the inability of the person with more advanced stages of AD to understand the diagnosis make communicating the diagnosis difficult [33]. The challenge lies in finding the right time and balancing the values of preserving autonomy, beneficence and non-maleficence [32].

Disclosing a risk status might not carry such a burden, since it is not known for sure how likely MCI turns into dementia [37]. But it is also very likely, that after disclosing a risk assessment, the status of the person changes, i.e. the person becomes a “patients-in-waiting” as described in the context of predictive genetic information [38]. This generates a more sensitive question about *when and how* to disclose a diagnosis or risk assessment, bearing in mind the person’s right (not) to know and the uncertainty of the risk information. Some first studies indicate that positive biomarker testing is potentially harmful to the tested person, by which the person might unnecessarily develop anxiety, fear, depression and stigmatization [6, 39]. In that sense, risk information might generate unnecessary psychological burden. Although the course of MCI cannot be predicted with certainty, a study conducted in the US reported that stigma is associated with an early stage or even a preclinical stage [40]. This can lead to a denial of symptoms, distancing one’s self from society as well as preventing help seeking behavior [41]. Additionally, current studies specifically addressing ‘MCI due to AD’ communication indicate the occurrence of suicidal thoughts [42]. Knowing the risk might arouse strong emotional, overwhelming reactions and reinforce the idea of suicide^10^ among the tested persons and family caregivers [41]. Such uncertainty in the progress of the disease, unnecessary anxiety and stigmatization might decrease the beneficial use of predictive information [6].

The possibility of knowing in advance might be argued to enhance one’s autonomy. The timing here does not seem to be so challenging as the period might be many years ahead which can increase ‘preparedness’. Besides, it might be advantageous to avoid others (i.e. family members) deciding later for the person, which carries the possibility of overrunning the person’s own interests. An interview study conducted with persons tested for risk for developing a dementia and diagnosed with an early dementia as well as their caregivers reports potential long-term effects such as allowing both the tested person and their family members for life planning, arrangement of advance care directives, getting support, starting with medication and modification of risk factors [41].

Another relevant level is the motivation to be tested. The motivation for receiving a diagnosis of a disease, which is already clearly symptomatic, brings often relief, even if the therapeutic options are limited. Especially in dementia, a motivation for getting a diagnosis can be to plan one’s future [36]. Therefore, detailed counseling after receiving the diagnosis seems to be a priority from an ethical point of view.

In risk prediction, the motivations are more diverse and ambivalent. In some cases, such as predictive genetic testing, motivation can be guided by wrong expectations about preventive or therapeutic measures as well as relieving uncertainty. For instance, although carrying a certain type of gene does not mean that the person will ultimately develop the disease [48], it is acknowledged as reliable providing high certainty to the tested person [49], and as having implications for planning family life concerning reproduction and informing the offspring [50]. Also, the general missing risk literacy can lead to wrong assumptions about how common and how high the general risk is [51]. Therefore, counseling in advance, before testing seems to be very crucial from an ethical point of view.

From this analysis, we hypothesize that, compared to disclosing a diagnosis of dementia, communicating a diagnosis of MCI and risk status for dementia in a preclinical stage require other kinds of sensitivities, counseling procedures and even multi-professional involvement. The challenge occurs with defining an asymptomatic, or minimally symptomatic, person at risk status due to positive biomarker results related to AD, of which, however, the diagnostic or predictive value is unclear [33].

#### Addressing the unsolved issues: approaches to deal with the gap

According to our view, (inter)national efforts should strive for specific guidelines on counseling, communicating and disclosing dementia risk results. To ensure that the needs of those potentially affected are met, empirical outcomes of effects of risk perception, risk communication strategies and psychosocial support should inform any guideline developments [2]. In case of risk assessment, this would include therefore not only patient with dementia or representatives of patient advocacy, but also asymptomatic laypersons, who represent the potential candidates.

In the following, we refer to the recommendations made by the Ad Hoc Working Group in the German Stakeholder Conference in 2018 [24]. They indicate a reasonable and an ethically profound way to fill the gap for two reasons. First, as this stakeholder conference was organized as a multi-professional and multidisciplinary discourse event, it was not dominated from the beginning by any particular professional interests as it might be a practical limitation of clinical guidelines. As a discourse event, it also allowed to consider culturally specific issues that might be overseen in a purely clinical-medical oriented procedure. Second, this conference made already some basic, but yet helpful, starting points, from which future developments can benefit. Overall, as predictive information has both harmful and beneficial aspects, the affected persons and their family members’ right to (or not to) know should be protected during the counseling process. Legal frameworks addressing the right not to know focused until now mainly on genetic testing. Hence, it is important, to enlarge this principle to any other predictive type of testing consequently. In practice, this means that risk prediction should neither be disclosed routinely nor be allowed in direct-to-consumer manner. Healthcare practitioners should be trained, should pay attention to the tested persons’ needs and preferences, and should act accordingly.

The Ad Hoc group [24] also reflected on how the testing practice should be embedded in a larger public discourse on dementia. If the public image of dementia remains stigmatic and stereotypical, the psychosocial implications will also be much more problematic. Such an unfavorable image of the disease is aimed to be eliminated by developing dementia-friendly strategies. The often-cited aim of dementia-friendly societies should indicate not only treatment and prevention strategies as the current policies mostly focus on, but also acceptance of the disease as a possible phase of the human life in the old age. However, it remains, yet, in many parts as ideal or rhetoric, and hence far from reality. Nonetheless, there are some hopeful approaches, which should be developed in parallel to such testing practice.

The proposed pre- and post-counseling should be multidisciplinary (i.e. not only medical, but also social and psychological aspects must be covered), and the information provided should be comprehensive and easily accessible in lay language. The Ad Hoc group also suggested that the offering for counseling should be legally mandatory [24]. Such an approach avoids a way too paternalistic attitude. Finally, further research on the harms and benefits of predicting dementia is needed, which can be used to address evidence-based, applicable guidelines and approaches for counseling [24].

The Risk Evaluation and Education of Alzheimer’s Disease (REVEAL) study was conducted with asymptomatic adults who had a parent with AD [52]. This study presents a counter-example to the concerns often raised for disclosing risk information: i.e. potential harmful effects, such as emotional distress, would outweigh the likely benefits. The REVEAL study showed that revealing the results of their APOE genotyping with genetic counseling did not lead to greater anxiety or depression compared to the non-disclosure group [52]. This study also indicates the importance of a structured disclosure process including pre- and post-counseling, but does not address in detail the different cultural and social backgrounds one might need to consider for a clinical practice. Therefore, we are skeptical to adopt these results directly to dementia prediction by other means than genetic and also in other cultural settings. Instead, it is crucial to have culturally embedded, cross-examined studies in order to understand how emotional, psychological, moral attitudes towards new technologies and prediction are shaped when people receive such risk status. Such encouragement on further culturally embedded research would help to develop responsive guidelines accordingly to the needs and preferences of those affected. Further scientific research is also needed on the use of biomarkers in predictive testing and diagnostics to avoid raising false hopes regarding their clinical certainty, for instance when a test result is negative [53]. Therefore, guidelines’ development should be accompanied by research exploring the experiences and expectations of affected persons in different cultural and health policy contexts.

### Limitations

Our analysis has some limitations, of course. It is restricted to only three Western countries among many other countries that are developing national dementia strategies. We might have therefore overseen other local guidelines that already address dementia prediction in a sufficient way. However, our ongoing literature search did not indicate such a development. We referred to governmental websites to acquire knowledge concerning the development and adoption of the national dementia plans and to practice (clinical) guidelines offered by nationwide and acknowledged societies, associations, working groups, which are the most comprehensive and well-adopted ones, but not to recommendations from local professional societies. The analysis of these three countries shows that current policies seem to neglect or, at least, not sufficiently address the issue of dementia prediction yet. Our ethical analysis is also rather on a more general, preliminary level and does not provide in itself a detailed approach concerning how the guidelines should be. We rather indicate some procedural and general aspects that should be discussed and be refined by a larger community of ethicists, practitioners and stakeholders.

## Conclusions

In this paper, we identified the challenges in disclosing a prediction of dementia and examined exemplarily three countries’ dementia strategies and clinical guidelines. Unfortunately, the guidelines do not address the new dementia developments sufficiently. Although the challenges and concerns regarding the disclosing of a diagnosis and even risk prediction have been raised, we were unable to identify any *specific clinical guidelines* regarding how to disclose the risk status. The need to develop empirically driven guidelines focusing specifically on disclosing risk information and risk communication strategies must therefore be addressed. The German Stakeholder Conference presents us a good starting point to the applicability of including stakeholders, which could be a step to meet the expectations of the affected persons and balancing them with practitioners’ concerns and to develop empirically-driven guidelines, as it is also the direction in Canada.

## Supplementary information

**Additional file 1: Table S1.** Countries with national dementia plans. **Table S2.** Countries developing national dementia plans.

## Data Availability

Not applicable.

## Abbreviations

AA: Alzheimer’s Association
AD: Alzheimer’s disease
APOE: Apolipoprotein E
APOE4: Apolipoprotein E4
APP: Amyloid Precursor Protein
CCCDTD: The Canadian Consensus Conference on the Diagnosis and Treatment of Dementia
DGN: The German Society for Neurology
DGPPN: The German Society for Psychiatry, Psychotherapy, Psychosomatics and Neurology
GMA: The German Medical Association
MAID: Medical Assistance in Dying
MCI: Mild Cognitive Impairment
NDP: National Dementia Plan
NIA: The National Institute on Aging
NIH: The National Institutes of Health
PGES: The Standford Program in Genomics, Ethics, and Society
PSEN1: Presenilin-1
PSEN2: Presenelin-2
REVEAL: The Risk Evaluation and Education of Alzheimer’s Disease
WHO: World Health Organization

## Footnotes

1 An additional table shows which countries and territories have already adopted a national plan and which countries are currently developing one [see Additional file 1].

2 There are important ongoing research projects in these three countries. For USA, please see: Anti-Amyloid Treatment in Asymptomatic Alzheimer’s Disease (A4) (https://a4study.org/about/); Risk Evaluation and Education for Alzheimer’s Disease – The Study of Communicating Amyloid Nueroimagining (REVEAL-SCAN) (https://clinicaltrials.gov/ct2/show/NCT02959489). For Canada, please see: The Comprehensive Assessment of Neurodegeneration and Dementia (COMPASS-ND) Study (http://ccna-ccnv.ca/compass-nd-study/). For Germany, please see: [Ethical and Legal Framework for the Prediction of Alzheimer’s Dementia] https://www.ceres.uni-koeln.de/forschung/projekte/predadqol/; AgeWell.de (https://www.gesundheitsforschung-bmbf.de/de/agewell-was-hilft-bei-demenz.php).

3 It should be noted that the recommendations generated by CCCDTD induced evidence-based guidelines on AD and other dementias [16]. The last updated recommendations and guidelines were published in 2012 [18].

4 For further information, please see: https://www.wegweiser-demenz.de/allianz-fuer-menschen-mit-demenz/die-allianz.html

5 The involved parties were neurologists, psychologists, care professionals, social workers, ethicists and representatives from patient advocacy groups, churches and health insurance associations. It was organized within a research project (PI: Silke Schicktanz, Scott Stock Gissendanner) and, therefore, independent from the existing policy structures, but the representatives often had a direct link to other policy settings.

6 For further information, see the English translation of the statement at http://praediadem.de/.

7 It should be noted that, as Table 1 illustrates as well, regarding disclosing a diagnosis of dementia or MCI, there is a consensus on the importance of multi-professional, continuous counseling, which includes providing information on diagnostic uncertainty, treatment, care services and social support groups, etc. to the tested person and to a family member. However, these recommendations basically stay at a very general level.

8 There are also studies showing that there is a public willingness to know one’s risk of developing dementia [27, 28].

9 Apart from the emphasized need for pre- and post-genetic counseling for diagnostic and predictive genetic testing [12, 13, 17, 18, 23], we could not come across any pre-counseling procedure concerning diagnostic and predictive tests for dementia. A counseling process is only mentioned, as also shown on Table 1, after having a diagnosis [10, 17, 22]. As with the growing interest on biomarkers, it is recommended that if the persons (and their families) would ask for biomarkers, the physician should explain them that there are no acceptable biomarkers yet [11, 18].

10 How strong the connection is between receiving an early diagnosis and developing suicidal thoughts remains a challenging empirical question. However, this option must be part of the extended ethical discussion on suicide and active euthanasia debate. Considering suicide as a pre-emptive option because of a very likely dementia disease might be seen as a rational choice and if so, should be respected [43]. However, due to the uncertainty of the prognosis of the disease based on the biomarker tests, pre-emptive suicide has not arguable validity [44]. The empirically observed emphasis on considering suicide as an option by lay persons [41, 45] could be read more generally as an emphasis on self-determination and wish for control over how to end one’s own life. This opinion seems strongly associated with the image of the disease, namely that dementia is generally conceptualized as leading to losing one’s control over oneself. However, this imagination and anticipation is also fuelled by stigma and problematic stereotypes. Hence, it remains an open question whether the international movement and increasing trend towards acceptance of suicide and active euthanasia is an ethically appropriate framing. On the other hand, it is questionable, whether reasons for voluntary suicide should be morally or even legally classified in good/bad categories in modern liberal societies. Assisted suicide based on the practical involvement of doctors remains here another challenge. In Germany, the right to assisted suicide was recently categorised as a fundamental human right [46] and in Canada, the Parliamentary Special Joint Committee on Physician-Assisted Dying suggested including mental illnesses in medical assistance in dying (MAID) [47]. Inevitably, such legal movements might also shape the public image of dementia as dramatic and, therefore, may influence individual strategies to cope with dementia. This needs an intense ethical, empirically informed examination, which has not yet sufficiently undertaken.

## References

[1] Sperling RA, Aisen PS, Beckett LA, Bennett DA, Craft S, Fagan AM (2011). Toward defining the preclinical stages of Alzheimer’s disease: recommendations from the National Institute on Aging-Alzheimer’s association workgroups on diagnostic guidelines for Alzheimer’s disease. Alzheimers Dement.

[2] Schicktanz S, Schweda M, Ballenger JF, Fox PJ, Halpern J, Kramer JH (2014). Before it is too late: professional responsibilities in late-onset Alzheimer’s research and pre-symptomatic prediction. Front Hum Neurosci.

[3] Leibing A (2018). Situated prevention: framing the ‘new dementia’. J Law Med Ethics.

[4] World Health Organization (2019). Risk reduction of cognitive decline and dementia: WHO guidelines.

[5] Molinuevo JL, Cami J, Carné X, Carrillo MC, Georges J, Isaac MB (2016). Ethical challenges in preclinical Alzheimer’s disease observational studies and trials: results of the Barcelona summit. Alzhiemers Dement.

[6] Schermer MHN, Richard E (2019). On the reconceptualization of Alzheimer’s disease. Bioethics.

[7] World Health Organization (2018). Towards a Dementia plan: a WHO guide.

[8] U.S. Department of Health and Human Services (2018). National plan to address Alzheimer‘s disease: 2018 update.

[9] The Alzheimer’s Association: Diagnostic Criteria and Guidelines. https://www.alz.org/research/for_researchers/diagnostic-criteria-guidelines. Accessed 31 Aug 2018.

[10] Gerontological Society of America (2017). KAER toolkit: A 4-Step process to detecting cognitive impairment and earlier diagnosis of Dementia: approaches and tools for primary care providers.

[11] Petersen RC, Lopez O, Armstrong MJ, Getchius TSD, Ganguli M, Gloss D (2018). Practice guideline update summary: mild cognitive impairment: report of the guideline development, dissemination, and implementation Subcommittee of the American Academy of neurology. Neurology.

[12] Relkin NR, Kwon YJ, Tsai J, Gandy S (1996). The National Institute on Aging/Alzheimer’s association recommendations on the application of Apolipoprotein E genotyping to Alzheimer’s disease. Ann N Y Acad Sci.

[13] McConnell LM, Koenig BA, Greely HT, Raffin TA, Members of the Alzheimer Disease Working Group of the Stanford Program in Genomics, Ethics, and Society (1999). Genetic testing and Alzheimer disease: recommendations of the Stanford program in genomics, ethics, and society. Genet Test.

[14] Goldmann JS, Hahn SE, Catania JW, LaRusse-Eckert S, Butson MB, Rumbaugh M (2011). Genetic Counselling and testing for Alzheimer disease: joint practice guidelines of the American College of Medical Genetics and the National Society of genetic counselors. Genet Med.

[15] Public Health Agency of Canada (2018). Conference Report: National Dementia Conference: Inspiring and Informing a National Dementia Strategy for Canada.

[16] Public Health Agency of Canada (2019). A National Dementia Strategy for Canada: Together We Aspire.

[17] Patterson C, Gauthier S, Bergman H, Cohen C, Feightner JW, Feldman H (2001). The recognition, assessment and Management of Dementing Disorders: conclusions from the Canadian consensus conference on dementia. Can J Neurol Sci.

[18] Gauthier S, Patterson C, Chertkow H, Gordon M, Herrmann N, Rockwood K (2012). Recommendations of the 4th Canadian consensus conference on the diagnosis and treatment of dementia (CCCDTD4). Can Geriatr J.

[19] Pimlott NJG, Persaud M, Drummond N, Cohen CA, Silvius JL, Seigel K (2009). Family Physicians and Dementia in Canada: Part 2. Understanding the Challenges of Dementia Care. Can Fam Physician.

[20] Toward Optimized Practice (TOP) Cognitive Impairment CPG Committee (2017). Cognitive impairment: symptoms to diagnosis clinical practice guideline.

[21] Federal Ministry for Family Affairs, Senior Citizens, Women and Youth and Federal Ministry of Health (2018). Alliance for People with Dementia: Report on the Implementation of the Agenda of the Alliance for People with Dementia 2014-2018.

[22] Deuschl G, Maier W, Zusammenarbeit mit der deutschen Alzheimer Gesellschaft e.V (2016). S3-Leitlinie “Demenzen” Langversion. Deutsche Gesellschaft für Psychiatrie und Psychotherapie, Psychosomatik und Nervenheilkunde (DGPPN), Deutsche Gesellschaft für Neurologie (DGN).

[23] The German Medical Association [Bundesärztekämmer]. Stellungnahme zum Umgang mit Prädiktiven Tests auf das Risiko für die Alzheimer Krankheit. Deutsches Ärzteblatt. 2018. 10.3238/arztebl.2018.sn_alzheimer01.

[24] Ad Hoc Working Group in the German Stakeholder Conference on Conflicts in Predictive Dementia Diagnostics (2018). Consensual Position Statement.

[25] Kolata G. A blood test for Alzheimer’s? It’s coming, Scientists Report: The New York Times; 2019. https://www.nytimes.com/2019/08/01/health/alzheimers-blood-test.html. Accessed 26 Mar 2020.

[26] Rawlinson K (2019). Alzheimer’s blood test could predict onset up to 20 years in advance. The Guardian.

[27] Alzheimer Europe (2011). The value of knowing: findings of Alzheimer Europe’s five country survey on public perceptions of Alzheimer’s disease and views on the value of diagnosis.

[28] Luck T, Luppa M, Sieber J, Schomerus G, Werner P, König HH (2012). Attitudes of the German population toward early diagnosis of dementia – results of a representative telephone survey. PLoS One.

[29] Schweda M, Kögel A, Bartels C, Wiltfang J, Schneider A, Schicktanz S (2018). Prediction and early detection of Alzheimer’s dementia: professional disclosure practices and ethical attitudes. J Alzheimers Dis.

[30] Evans JP, Skrzynia C, Burke W (2001). The complexities of predictive genetic testing. BMJ.

[31] Werner P, Karnieli-Miller O, Eidelman S (2013). Current knowledge and future directions about the disclosure of dementia: a systematic review of the first decade of the 21st century. Alzheimers Dement.

[32] Pinner G, Bouman WP (2002). To tell or not to tell: on disclosing the diagnosis of dementia. Psychogeriatr.

[33] Gauthier S, Leuzy A, Racine E, Rosa-Neto P (2013). Diagnosis and Management of Alzheimer’s disease: past, present and future ethical issues. Prog Neurobiol.

[34] Fisk JD, Beattie BL, Donnelly M, Byszewski A, Monar FJ (2007). Disclosure of the diagnosis of dementia. Alzheimers Dement.

[35] Iliffe S, Robinson L, Brayne C, Godman C, Rait G, Manthorpe J (2009). Primary care and dementia : 1. Diagnosis, screening and disclosure. Int J Geriatr Psychiatry.

[36] Van den Dungen P, van Kuijk L, van Marwijk H, van der Wouden J, van Charante EM, van der Horst H (2014). Preferences regarding disclosure of a diagnosis of dementia: a systematic review. Int Psychogeriatr.

[37] Ganguli M, Snitz BE, Saxton JA, Chang CCH, Lee CW, Bilt JV (2011). Outcomes of mild cognitive impairment by definition: a popular study. Arch Nuerol.

[38] Timmermanns S, Buchbinder M (2010). Patients-in-waiting: living between sickness and health in the genomics era. J Health Soc Behav.

[39] Le Couteur DG, Doust J, Creasey H, Brayne C (2013). Political drive to screen for pre-dementia: not evidence based and ignores the harms of diagnosis. BMJ.

[40] Johnson R, Harkins K, Cary M, Sankar P, Karlawish J (2015). The relative contributions of disease label and disease prognosis to Alzheimer’s stigma: a vignette-based experiment. Soc Sci Med.

[41] Lohmeyer L, Alpinar-Sencan Z, Schicktanz S. Attitudes towards prediction and early diagnosis of late-onset dementia: a comparison of tested persons and family caregivers. Aging Ment Health. 2020. 10.1080/13607863.2020.1727851 [Epub ahead of print]. 10.1080/13607863.2020.1727851 32091238

[42] Milne R, Bunnik E, Diaz A, Richard E, Badger S, Gove D (2018). Perspectives on communicating biomarker-based assessments of Alzheimer’s disease to cognitively healthy individuals. J Alzheimers Dis.

[43] Davis DS (2014). Alzheimer’s disease and pre-emptive suicide. J Med Ethics.

[44] Dresser R (2014). Pre-Emptive suicide, precedent autonomy and preclinical Alzheimer’s disease. J Med Ethics.

[45] Alpinar-Sencan Z, Lohmeyer L, Schicktanz S (2020). Planning later life with dementia: comparing family caregivers’ perspectives on biomarkers with laypersons’ attitudes towards genetic testing of dementia prediction. New Genet Soc.

[46] The Federal Constitutional Court (2020). Criminalisation of assisted suicide services unconstitutional.

[47] Parliament of Canada (2016). Medical assistance in dying: a patient centered approach. Report of the special joint committee on physician-assisted dying.

[48] Alper JS, Beckwith J (1998). Distinguishing genetic from nongenetic medical tests: some implications for antidiscrimination legislation. Sci Eng Ethics.

[49] Dufrasne S, Roy M, Galvez M, Rosenblatt DS (2011). Experience over fifteen years with a protocol for predictive testing for Huntington disease. Mol Genet Metab.

[50] Arribas-Ayllon M (2011). The ethics of disclosing genetic diagnosis for Alzheimer’s disease: do we need a new paradigm?. Br Med Bull.

[51] Wegwarth O, Gigerenzer G, Goerling U, Mehnert A (2018). The barrier to informed choice in Cancer screening: statistical illiteracy in physicians and patients. Psycho-oncology. Recent results in Cancer research.

[52] Green RC, Roberts S, Cupples LA, Relkin NR, Whitehouse PJ, Brown T (2009). Disclosure of APOE genotype for risk of Alzheimer’s disease. N Engl J Med.

[53] Grill JD, Cox CG, Kremen S, Mendez MF, Teng E, Shapira J (2017). Patient and caregiver reactions to clinical amyloid imaging. Alzheimers Dement.

