# Supplementary material for: Addressing ethical challenges of disclosure in dementia prediction: limitations of current guidelines and suggestions to proceed
Source: BMC Med Ethics. 2020 May 11;21:33. doi: 10.1186/s12910-020-00476-4 (PMC7216419; doi:10.1186/s12910-020-00476-4)
Supplement: Supplementary file 1 — Additional file 1: Table S1. Countries with national dementia plans. Table S2. Countries developing national dementia plans. [file 12910_2020_476_MOESM1_ESM.pdf]

**Table 1[1]: Countries with national dementia plans:**					
Australia	Czech Republic	Israel	Malta	Puerto Rico	UK
Austria	Denmark	Italy	Mexico	Republic of Korea	USA
Chile	Finland	Japan	Netherlands	Slovenia	
Costa Rica	Greece	Luxembourg	New Zealand	Switzerland	
Cuba	Indonesia	Macau SAR	Norway	TADA Chinese Taipei	

**Table 2[1]: Countries developing national dementia plans:**					
Argentina	Bonaire	Croatia	India	Panama	Sri Lanka
Bangladesh	Bosnia-Herzegovina	Dominican Republic	Lesotho	Peru	Sweden
Barbados	Brunei	El Salvador	Malaysia	Portugal	Vietnam
Brazil	Canada[2]	Germany	Mauritius	South Africa	Uruguay
Bolivia	Columbia	Kenya	Nigeria	Spain	

1 We refer to the 2018 data obtained from Alzeimer's Disease International (see: https://www.alz.co.uk/dementia-plans. Accessed 14 Sep 2018)
2 Quite recently, in June 2019, Canada developed its national dementia strategy. Since the data was obtained from Alzheimer Disease International in 2018, Canada is grouped under the countries developing national strategies.
